# Double-blind randomized study on the myeloprotective effect of melatonin in combination with carboplatin and etoposide in advanced lung cancer

**DOI:** 10.1038/sj.bjc.6690463

**Published:** 1999-06

**Authors:** M Ghielmini, O Pagani, J de Jong, S Pampallona, A Conti, G Maestroni, C Sessa, F Cavalli

**Affiliations:** Servizio Oncologico Cantonale, Ospedale San Giovanni, 6500 Bellinzona, Switzerland; Istituto Patologico Cantonale, 6600 Locarno, Switzerland; Formed, Statistics for Medecine, 1983 Evolène, Switzerland

**Keywords:** myelotoxicity, chemotherapy, hormones, melatonin

## Abstract

A significant myeloprotective effect of melatonin in mice treated with etoposide, cyclophosphamide or carboplatin has been reported. The present study was designed to evaluate if the same effect could be observed in patients receiving chemotherapy. Twenty previously untreated patients with inoperable lung cancer received two cycles of carboplatin (given at area under the curve 5 by the Calvert formula) on day 1 and etoposide (150 mg m^−2^ i.v.) on days 1–3 every 4 weeks. Melatonin 40 mg or placebo (double-blind) was given orally in the evening for 21 consecutive days, starting 2 days before chemotherapy. Patients were randomized to receive melatonin either with the first or the second cycle. Complete blood cell count with differential was done three times per week for 3 weeks. The median age of the cohort was 60 years (range 42–69), 16 patients had non-small cell and four patients small-cell lung cancer, 12 stage III and eight stage IV disease. In a multivariate analysis including age, sex, diagnosis, stage, performance status, doses of carboplatin and etoposide, and concomitant treatment with melatonin or placebo, the haematological parameters – depth and duration of toxicity for haemoglobin, platelets and neutrophils (ANC) – were not significantly different between cycles with/without melatonin. The mean ANC nadir and the mean number of days with ANC < 0.5 × 10^9^ l^−1^ were 0.5 × 10^9^ l^−1^ and 2.5 days, respectively, with/without melatonin. We concluded that, in patients with lung cancer, melatonin given orally at a dose of 40 mg per day for 21 days in the evening, does not protect against the myelotoxic effect of carboplatin and etoposide. © 1999 Cancer Research Campaign


*British Journal of Cancer* (1999) **80**(7), 1058–1061
© 1999 Cancer Research Campaign
Article no. bjoc.1998.0463

### Double-blind randomized study on the myeloprotective effect of melatonin in combination with carboplatin and etoposide in advanced lung cancer

**M Ghielmini[1], O Pagani[1], J de Jong[1], S Pampallona[3], A Conti[2], G Maestroni[2], C Sessa[1] and F Cavalli[1]**

[1]Servizio Oncologico Cantonale, Ospedale San Giovanni, 6500 Bellinzona, Switzerland; [2]Istituto Patologico Cantonale, 6600 Locarno, Switzerland; [3]Formed, Statistics for Medecine, 1983 Evolène, Switzerland

**Summary** A significant myeloprotective effect of melatonin in mice treated with etoposide, cyclophosphamide or carboplatin has been reported. The present study was designed to evaluate if the same effect could be observed in patients receiving chemotherapy. Twenty previously untreated patients with inoperable lung cancer received two cycles of carboplatin (given at area under the curve 5 by the Calvert formula) on day 1 and etoposide (150 mg $m^{-2}$ i.v.) on days 1–3 every 4 weeks. Melatonin 40 mg or placebo (double-blind) was given orally in the evening for 21 consecutive days, starting 2 days before chemotherapy. Patients were randomized to receive melatonin either with the first or the second cycle. Complete blood cell count with differential was done three times per week for 3 weeks. The median age of the cohort was 60 years (range 42–69), 16 patients had non-small cell and four patients small-cell lung cancer, 12 stage III and eight stage IV disease. In a multivariate analysis including age, sex, diagnosis, stage, performance status, doses of carboplatin and etoposide, and concomitant treatment with melatonin or placebo, the haematological parameters – depth and duration of toxicity for haemoglobin, platelets and neutrophils (ANC) – were not significantly different between cycles with/without melatonin. The mean ANC nadir and the mean number of days with ANC $< 0.5 \times 10^9$ $l^{-1}$ were $0.5 \times 10^9$ $l^{-1}$ and 2.5 days, respectively, with/without melatonin. We concluded that, in patients with lung cancer, melatonin given orally at a dose of 40 mg per day for 21 days in the evening, does not protect against the myelotoxic effect of carboplatin and etoposide.

**Keywords:** myelotoxicity; chemotherapy; hormones; melatonin

Escalating the dose of chemotherapy could increase response rate and, possibly, survival in some tumour types (Gurney et al, 1993), usually with a higher degree of haematological toxicity. The recent spread of dose-intensive treatments has stimulated the research of possible means to reduce degree and/or duration of haematological toxicities. Some of these methods, such as administration of haemopoietic growth factors, granulocyte colony-stimulating factor (G-CSF), or granulocyte–macrophage CSF (GM-CSF), or transfusion of autologous blood stem cells, have already entered common daily practice (Canellos, 1994). Other methods are still investigational, such as new growth factors (stem cell factor or FLT-3 ligand), concomitant administration of potentially myeloprotective agents (amifostine and seraspenide) (Capizzi et al, 1993; Genevay et al, 1996), bone marrow inhibitors (MIP-1α) or transfection of stem cells with multidrug resistant (MDR) genes (Jelinke et al, 1996).

A myeloprotective and immuno-enhancing effect of the pineal neuro-hormone melatonin in in-vitro and in-vivo models has been recently described (Maestroni et al, 1994*b*). In physiological conditions, melatonin is secreted by the pineal gland with a circadian rhythm and it has recently received renewed interest because of some demonstrated (induction of sleep), or claimed, properties (anti-aging or anti-tumour activity) (Arendt, 1996; Brzezinski, 1997). In medical oncology, melatonin has been given either in combination with interleukin-2 (IL-2) or interferon to patients with melanoma and renal cell cancer, and alone or in association with chemotherapy in patients with lung cancer or brain metastasis (Lissoni et al, 1989; Lissoni et al, 1992; Aldeghi et al, 1994; Neri et al, 1994).

In tumour-bearing mice treated with cyclophosphamide, etoposide or carboplatin, the association of melatonin with chemotherapy was shown to abrogate leucopenia and thrombocytopenia without affecting anti-tumour activity (Maestroni et al, 1994*b*). The myeloprotective effect was more pronounced when melatonin was given concomitantly rather than after chemotherapy. In vitro experiments on haemopoietic stem cells suggested that this effect was obtained through inhibition of apoptosis, mediated by a mechanism involving T-helper cells and GM-CSF (Maestroni et al, 1994*a*). This effect seems to be dependent on opioid cytokines released by T-helper cells after stimulation with melatonin, and is similar to the effect of both IL-4 and dynorphin (Maestroni et al, 1996).

The present study was performed to verify if the myeloprotective effect of melatonin observed in mice could be reproduced in humans receiving chemotherapy. Previously untreated patients with inoperable lung cancer were scheduled to receive two cycles of carboplatin and etoposide, at dosages known to induce response but at the price of a severe myelosuppression. Patients were randomly assigned to receive either melatonin or placebo at the first cycle, with cross over to the other treatment at the second cycle.

*Received 28 July 1998*
*Revised 27 November 1998*
*Accepted 4 December 1998*

*Correspondence to:* M Ghielmini

Presented in part at the annual meeting of the American Society of Clinical Oncology 1997.

### MATERIALS AND METHODS

Eligibility criteria included age of 18–70 years, histological or cytological diagnosis of non-small-cell (NSCLC) or small-cell (SCLC) lung cancer not amenable to surgery or radiotherapy, no previous chemotherapy or radiotherapy, normal blood counts and normal serum creatinine. In case of undifferentiated small-cell histology, or in case of bone metastases, evidence of uninvolved bone marrow by trephine biopsy was required. Concomitant treatment with radiotherapy, steroids, haemopoietic growth factors or investigational agents was not allowed. Patients were asked for written informed consent and the trial was approved by the local ethical authorities.

Chemotherapy consisted of two cycles of carboplatin (5 areas under the curve (AUC) by the Calvert formula) (Crinò et al, 1995) on day 1 and etoposide (150 mg $m^{-2}$ intravenously (i.v.)) on days 1–3 given in the morning every 4 weeks. The doses administered in the first cycle had to be repeated in the second cycle without any dose adjustment, provided the creatinine clearance was in the normal range. Patients showing non-haematological toxicity ≥ grade 3 according to NCI–CTC criteria or haematological toxicity leading to hospitalization (haemorrhage or neutropenic fever) at the first cycle, went off study.

Melatonin was provided by Helsinn Chemicals (Biasca, Switzerland) as gelatin capsules containing 40 mg of the active substance in a corn oil suspension. Placebo capsules had the same aspect and content except for melatonin. To assure stability, capsules were kept in a –20°C freezer; patients were instructed to keep them in the refrigerator. During the treatment, melatonin or placebo had to be taken orally in the evening (20:00 h), starting 2 days before chemotherapy for a total of 21 consecutive days. Treatment was coded, and capsules were kept and distributed by the research nurse who was in charge of the registration and randomization; neither the nurse, the patient nor the investigator were aware of the nature of the content. Patients were randomly assigned to receive melatonin or placebo at the first cycle, with cross over to the other treatment at the second cycle, to avoid that results could be influenced by a cumulation of myelotoxicity.

Patients were on study up to 4 weeks after the second cycle. During the study, patients had weekly clinical controls, with complete blood cell count with differential three times a week until complete haematological recovery (white blood count > $4 \times 10^9$ $l^{-1}$ and platelets > $100 \times 10^9$ $l^{-1}$). Chemistry, including creatinine, liver function tests (LFT), glucose and PT were measured weekly. Formal disease assessment was planned after the two cycles, or before if clinically indicated. After the first two cycles, patients could be treated according to the physician's preference. For those wishing it, unblinded melatonin was available from the 3rd cycle on.

Statistical analysis was performed by analysis of covariance (ANCOVA) for repeated (paired) observations after confirming the appropriateness of the assumption of normal distribution. The regression models were calculated with age (as continuous variable), sex, diagnosis (NSCLC vs SCLC), stage (III vs IV), performance status (0 vs I), dose of carboplatin and etoposide (mg $m^{-2}$ as continuous variable) melatonin or placebo and order of melatonin administration (at 1st or 2nd cycle) as independent variables. A random effect for patients was also included in the model. Statistical analysis was performed with the package 'Stata' (Stata Corp., Texas, 1997).

The study has been performed without formal sample-size computations. With the available sample-size of 20, a paired *t*-test would have an 80% (or 90%) power to detect a change in a given haematological parameter of the order of 65% (or 75%) of the standard deviation of the observations (Machin and Campbell, 1987). Given the analytical approach used here, even smaller effect sizes might have been detected.

**Table 1** Characteristics of evaluable patients ($n$ = 20)

| | |
|---|---|
| Median age (years) | 60 |
| Range | 42–69 |
| Male/Female | 15/5 |
| Small-cell/non-small-cell | 4/16 |
| Stage | |
| III | 12 |
| IV | 8 |
| Performance status | |
| 0 | 13 |
| 1 | 7 |

**Table 2** Mean (± s.d.) doses of carboplatin and etoposide

| | Etoposide (mg $m^{-2}$) | Carboplatin (mg $m^{-2}$) | Carboplatin (AUC) |
|---|---|---|---|
| 1st cycle | 430 ± 36 | 373 ± 74 | 4.8 ± 0.5 |
| 2nd cycle | 430 ± 36 | 333 ± 78 | 5.2 ± 1.4 |
| Without melatonin | 430 ± 36 | 354 ± 75 | 5.3 ± 1.4 |
| With melatonin | 420 ± 44 | 348 ± 65 | 4.9 ± 0.1 |

### RESULTS

Twenty-eight patients entered the study and eight (three with SCCL and five with NSCCL) were not evaluable for the effect of melatonin because of discontinuation of treatment after the first cycle (disease progression: five patients; excessive toxicity: three patients, all haematological).

The characteristics of the 20 patients with evaluable first and second cycles are summarized in Table 1. Eight patients had distant metastases, without bone marrow involvement in all of them. Table 2 reports the doses of carboplatin and etoposide administered at the first and second cycle with/without melatonin. The doses were equivalent in all groups; in particular, the dose of carboplatin was superimposable if it was calculated according to surface area or to Calvert formula. The apparent raise at the second cycle in the dose of carboplatin if calculated according to AUC was due to a reduction of the mean value of the creatinine clearance from 108 ml $min^{-1}$ to 97 ml $min^{-1}$. In the multivariate analysis, the effect of carboplatin dose remained the same if calculated per mg $m^{-2}$ or according to AUC. The median dose of melatonin per day was of 0.6 mg $kg^{-1}$ per day (range 0.4–0.8).

Overall, chemotherapy was well-tolerated, despite the significant dosages of etoposide administered, and no patient needed erythrocyte or platelet transfusions. Two patients (one at the first cycle, given with melatonin and the other at the second cycle, without concomitant melatonin) had to be hospitalized because of febrile neutropenia.

The observed means and standard deviations of the haematological parameters of interest for both melatonin and placebo are

© 1999 Cancer Research Campaign

**Table 3** Haematological toxicity of the cycles with placebo or melatonin (mean ± s.d.)

| Melatonin | No | | *P* |
|---|---|---|---|
| Nadir ANC × $10^9$ $L^{-1}$ | 0.5 ± 0.4 | 0.5 ± 0.3 | 0.82 |
| Days ANC < 1.0 × $10^9$ $l^{-1}$ | 6.3 ± 4.6 | 5.9 ± 3.5 | 0.58 |
| Days anc < 0.5 × $10^9$ $l^{-1}$ | 2.5 ± 2.8 | 2.5 ± 2.8 | 0.20 |
| Nadir platelets × $10^9$ $l^{-1}$ | 121 ± 52 | 134 ± 54 | 0.97 |
| Days platelets < 100 × $10^9$ $l^{-1}$ | 1.5 ± 3.1 | 1.0 ± 2.3 | 0.52 |
| Nadir Hb g per 100 ml | 11.4 ± 1.4 | 11.5 ± 0.8 | 0.34 |

*P*-values vere estimated by the ANCOVA regression models.

reported in Table 3. The mean ANC nadir was $0.5 \times 10^9$ $l^{-1}$ and the mean number of days with ANC < $0.5 \times 10^9$ $l^{-1}$ was 2.5, both without and with melatonin; mean platelets nadir was $121 \times 10^9$ $l^{-1}$ without and $134 \times 10^9$ $l^{-1}$ with melatonin, and the number of days with platelets under $100 \times 10^9$ $l^{-1}$ was respectively 1.5 and 1.0. The ANCOVA models demonstrate the lack of significance of the small variations observed with the adjunction of melatonin (Table 3). The haematological toxicity of the second cycle was, as expected, slightly higher, but the difference was not statistically significant. In the regression analysis, the only variables significantly influencing haematological toxicity were age, which was associated with a prolongation of ANC nadir ($P$ = 0.03), and worse performance status, associated with a deeper ANC nadir ($P$ = 0.007).

No changes of PT, glucose or LFT were seen, while a 10% reduction in creatinine clearance was noted from the first to the second cycle, but not between cycles with or without melatonin. Ten of the 28 patients entered in the trial (36%) achieved partial or complete response.

### DISCUSSION

The purpose of this randomized, double-blind trial was to evaluate the myeloprotective effect of melatonin in patients receiving chemotherapy. The rationale of the trial was represented by the previous experimental work of some of the authors demonstrating a myeloprotective effect of melatonin in tumour-bearing mice and in haematopoietic clonogenic cells in vitro (Maestroni et al, 1994*a*, 1994*b*). To mimic the experimental situation we administered melatonin before, during and after chemotherapy, until the expected day of recovery from neutropenia, for a total of 21 days. The dose of melatonin selected (40 mg per day) is much higher than the one shown to have physiological activity on sleep troubles, and is associated with biological effects in the immune system (Brzezinski, 1997), but it is nearer to the one tested in animals to evaluate myeloprotection (1 mg $kg^{-1}$) and equal to the one used in previous trials in cancer patients (Lissoni et al, 1994).

In the present study, chemotherapy with carboplatin and etoposide was generally well-tolerated, with few subjective side-effects, and haematological toxicity in the range of what is usually observed. The overall response rate (36%) was as expected for this type and stage of disease (Ihde, 1992; Crinò et al, 1995), suggesting that melatonin did not affect anti-tumour activity.

Even though neutropenia and thrombocytopenia showed a high interpatient variability, the concomitant administration of melatonin did not significantly affect the pattern of myelotoxicity: the mean (± s.d.) of ANC nadir and the mean number of days with an ANC < $0.5 \times 10^9$ $l^{-1}$ were $0.5 \pm 0.4 \times 10^9$ $l^{-1}$ and $0.5 \pm 0.3 \times 10^9$ $l^{-1}$ and 2.5 ± 2.8 days in cycles with/without melatonin respectively.

Even though we used a dose lower than the one tested in animals (0.6 mg $kg^{-1}$ orally vs 1 mg $kg^{-1}$ s.c. in the mouse) we believe that the dose used should have been active. The ingestion of 40-mg capsules is, in fact, known to result in plasma concentrations much above the physiological ones for more than 8 h (Waldhauser et al, 1984; Aldhous et al, 1985), and which have been associated with some biological activities, such as activation of some lymphocyte subsets in cancer patients (Lissoni et al, 1994).

We chose to give melatonin in the evening, in order to maintain its physiological circadian rhythm, but this timing could have affected its myeloprotective effect; in fact, the administration of melatonin concomitantly to chemotherapy might have a higher myeloprotective effect through its antioxidant action. We can therefore not exclude that the same trial, if performed with melatonin given in the morning, or shortly before chemotherapy, could have given different results.

In conclusion, our work demonstrates the lack of a protective effect of melatonin, given orally at a dose of 40 mg per day for 21 days in the evening, on the haematological toxicity induced by carboplatin and etoposide in patients with lung cancer.

### REFERENCES

Aldeghi R, Lissoni P, Barni S, Ardizzoia A, Tancini G, Piperno A, Pozzi M, Ricci G, Conti A and Maestroni GJM (1994) Low-dose interleukin-2 subcutaneous immunotherapy in association with the pineal hormone melatonin as a first-line therapy in locally advanced or metastatic hepatocellular carcinoma. *Eur J Cancer* **30A**: 167–170

Aldhous M, Franey C, Wright J and Arendt J (1985) Plasma concentrations of melatonin in man following oral absorption of different preparations. *Br J Clin Pharmacol* **19**: 517–521

Arendt J (1996) Melatonin. *Br Med J* **312**: 1242–1243

Brzezinski A (1997) Melatonin in humans. *N Engl J Med* **336**: 186–195

Canellos GP (1994) High-dose therapy: here to stay or just visiting? *J Clin Oncol* **12**: 5–6

Capizzi RL, Scheffler BJ and Schein PS (1993) Amifostine-mediated protection of normal bone marrow from cytotoxic chemotherapy. *Cancer* **72**: 3495–3501

Crinò L, Clerici M, Figoli F, Carlini P, Ceci G, Cortesi E, Carpi A, Santini A, Di Costanzo F, Boni C, Meacci M, Corgna E, Darwish S, Scarcella L, Santucci A, Ballatori E and Tonato M (1995) Chemotherapy of advanced non-small-cell lung cancer: A comparison of three active regimens. A randomized trial of the Italian Oncology Group for Clinical Research (G.O.I.R.C.). *Ann Oncol* **6**: 347–353

Genevay MC, Mormont C, Thomas F and Berthier R (1996) The synthetic tetrapeptide AcSDKP protects cells that reconstitute long-term bone marrow stromal cultures from the effects of mafosfamide (Asta Z 7654). *Exp Hematol* **24**: 77–81

Gurney H, Dodwell D, Thatcher N and Tattersall MHN (1993) Escalating drug delivery in cancer chemotherapy: A review of concepts and practice – part 1. *Ann Oncol* **4**: 23–34

Ihde DC (1992) Chemotherapy of lung cancer. *N Engl J Med* **327**: 1434–1441

Jelinek J, Fairbairn LJ, Dexter TM, Rafferty JA, Stocking C, Ostertag W and Margison GP (1996) Long-term protection of hematopoiesis against the cytotoxic effects of multiple doses of nitrosourea by retrovirus-mediated expression of human 06-alkylguanine-DNA-alkyltransferase. *Blood* **87**: 1957–1961

Lissoni P, Barni S, Crispino S, Tancini G and Fraschini F (1989) Endocrine and immune effects of melatonin therapy in metastatic cancer patients. *Eur J Cancer Clin Oncol* **25**: 789–795

Lissoni P, Tisi E, Barni S, Ardizzoia A, Rovelli F, Rescaldani R, Ballabio D, Benenti C, Angeli M, Tancini G, Conti A and Maestroni GJM (1992) Biological and clinical results of a neuroimmunotherapy with inteleukin-2 and the pineal hormone melatonin as a first line treatment in advanced non-small cell lung cancer. *Br J Cancer* **66**: 155–158

Lissoni P, Barni S, Tancini G, Ardizzoia A, Ricci G, Aldeghi R, Brivio F, Tisi E, Rovelli F, Rescaldani R, Quadro G and Maestroni G (1994) A randomised study with subcutaneous low-dose interleukin 2 alone vs interleukin 2 plus the pineal neurohormone melatonin in advanced solid neoplasms other than renal cancer and melanoma. *Br J Cancer* **69**: 196–199

 *© 1999 Cancer Research Campaign*

Machin D and Campbell MJ (1987) *Statistical tables for the design of clinical trials*. Blackwell Scientific Publications: Oxford.

Maestroni GJ, Conti A and Lissoni P (1994*a*) Colony-stimulating activity and hemopoietic rescue from cancer chemotherapy compounds are induced by melatonin via endogenous interleukin 4. *Cancer Res* **54**: 4740–4743

Maestroni GJ, Covacci V and Conti A (1994*b*) Hematopoietic rescue via T-cell-dependent, endogenous granulocyte-macrophage colony-stimulating factor induced by the pineal neurohormone melatonin in tumour-bearing mice. *Cancer Res* **54**: 2429–2432

Maestroni JM, Hertens E, Galli P, Conti A and Pedrinis E (1996) Melatonin-induced T-helper cell hematopoietic cytokines resembling both interleukin-4 and dynorphin. *J Pineal Res* **21**: 131–139

Neri B, Fiorelli C, Moroni F, Nicita G, Paoletti MC, Ponchietti R, Raugei A, Santoni G, Trippitelli A and Grechi G (1994) Modulation of human lymphoblastoid interferon activity by melatonin in metastatic renal cell carcinoma. *Cancer* **73**: 3015–3019

Stata Corp (1997) Stata Statistical Software. Stata corporation: (Abstract)

Waldhauser F, Waldhauser M, Lieberman HR, Deng M-H, Linch D and Wurtman RJ (1984) Bioavailability of melatonin in humans. *Neuroendocrinology* **39**: 307–313

*© 1999 Cancer Research Campaign*
